# Serotype- and strain- dependent contribution of the sensor kinase CovS of the CovRS two-component system to *Streptococcus pyogenes *pathogenesis

**DOI:** 10.1186/1471-2180-10-34

**Published:** 2010-02-01

**Authors:** Venelina Sugareva, Regina Arlt, Tomas Fiedler, Catur Riani, Andreas Podbielski, Bernd Kreikemeyer

**Affiliations:** 1 University of Rostock, Medical Faculty, Inst. of Med. Microbiology, Virology and Hygiene, Dept. of Med. Microbiology and Hospital Hygiene, Schillingallee 70, 18055 Rostock, Germany

## Abstract

**Background:**

The *Streptococcus pyogenes *(group A streptococci, GAS) two-component signal transduction system CovRS has been described to be important for pathogenesis of this exclusively human bacterial species. If this system acts uniquely in all serotypes is currently unclear. Presence of serotype- or strain-dependent regulatory circuits and polarity is an emerging scheme in *Streptococcus pyogenes *pathogenesis. Thus, the contribution of the sensor kinase (CovS) of the global regulatory two-component signal transduction system CovRS on pathogenesis of several M serotypes was investigated.

**Results:**

CovS mutation uniformly repressed capsule expression and hampered keratinocyte adherence in all tested serotypes. However, a serotype- and even strain-dependent contribution on survival in whole human blood and biofilm formation was noted, respectively.

**Conclusions:**

These data provide new information on the action of the CovS sensor kinase and revealed that its activity on capsule expression and keratinocyte adherence is uniform across serotypes, whereas the influence on biofilm formation and blood survival is serotype or even strain dependent. This adds the CovRS system to a growing list of serotype-specific acting regulatory loci in *S. pyogenes*.

## Background

*Streptococcus pyogenes *(group A streptococcus, GAS) is an important and exclusively human pathogen, which causes a variety of diseases ranging from mild superficial infections to invasive life-threatening illnesses with high mortality rates [1-4]. Successful colonization and persistence within the host relies on sensing and responding to the changes in the environmental conditions. These responses are very often mediated by two-component signal transduction regulatory systems (TCS). The CovRS (also called CsrRS, [5]) system is one of 13 TCS in the GAS genome, which has been extensively studied, and for which a central role in growth and pathogenesis was found [6-8]. CovR represses either directly or indirectly about 15% of the genes in GAS [9-11], many of which represent important virulence factors. CovR acts as a negative regulator of hyaluronic acid capsule biosynthesis by repression of the hyaluronic acid capsule (*has*) operon [5]. Additional virulence genes influenced by CovRS include *ska *(encoding streptokinase), *sagA *(encoding streptolysin S), *sda *(encoding streptococcal DNase) and *speB *(encoding a cysteine protease) [11,12]. CovRS activity modulates the transcriptome during growth in human blood [13]. Furthermore, mutations in CovRS lead to strains with enhanced virulence in animal models of skin and soft tissue infections [8,9,12]. A paper by Trevino *et al*. published during the review of this work investigated the influence of CovS on the CovR-mediated repression of GAS virulence factor-encoding genes [14].

The first step in GAS infection is the adherence of GAS to epithelia of the skin and respiratory tract, a process that is intensively studied on the molecular level [15-17]. This phenomenon is supported by host extracellular matrix proteins, such as collagen and fibronectin. The mechanism of adherence is enabled mainly by specific adhesion components on the GAS surface commonly termed MSCRAMMs (for microbial surface components recognizing adhesive matrix molecules) [16], which are under the control of several single response regulators and several two-component systems. Furthermore, the expression profile of the GAS MSCRAMMs is time - and serotype-dependent [16].

The initial adhesion process of GAS to matrix protein coated or an uncoated surface essentially contributes to the biofilm formation, a novel described feature of many clinically important serotypes of GAS [17]. Former studies showed that CovRS regulation appears to be critical for biofilm formation [18]. Recently, studies on biofilm regulation revealed, that streptococcal regulator of virulence (Srv) is also required for biofilm formation [19].

Increasing evidence now suggests that many GAS virulence traits and even the polarity of transcriptional regulatory circuits are serotype- and sometimes strain-specific [20,21]. Consequently, the importance of serotype- or strain- dependent CovS contribution to *S. pyogenes *pathogenesis was investigated. The CovS sensor kinase part of the two-component system was inactivated by insertional mutagenesis in different M serotype GAS strains and the wild type and isogenic mutant pairs were subsequently tested for biofilm formation, capsule expression, survival in whole human blood, and adherence to keratinocytes.

## Methods

### Bacterial strains and culture conditions

M49 strain 591 is a skin isolate provided from R. Lütticken (Aachen, Germany). The M2, M6 and M18 serotypes GAS strains are clinical isolates obtained from the collection of the Centre of Epidemiology and Microbiology, National Institute of Public Health, Prague, Czech Republic, and have been previously described [22]. *E. coli *DH5α was used as the host for plasmid constructions and was grown at 37°C with shaking in Luria broth. The GAS strains were cultured in static Todd-Hewitt broth (THB, Invitrogen) supplemented with 0.5% yeast extract (THY) at 37°C under a 5% CO_2_-20% O_2 _atmosphere without agitation. Erythromycin in a final concentration of 300 μg/ml for *E. coli *and 5 μg/ml for GAS was used for selection and maintenance of the mutants.

### Standard DNA techniques

Genomic DNA from GAS strains was isolated by DNeasy blood and tissue kit (Qiagen) according to the manufacturer's recommendations. Plasmid DNA manipulations, transformation of *E. coli *and GAS were performed as described previously [23]. *S. pyogenes *competent cells were prepared in the presence of glycin, mutanolysin and hyaluronidase, as follows: *S. pyogenes *was grown overnight in 10 ml THY broth supplemented with 20 mM glycin, then 5 ml of the pre-culture was added to 45 ml of THY supplemented with glycine (20 mM) and mutanolysin (10 U/ml) for overnight incubation. Cells were harvested by centrifugation at 3000 rpm, 4°C for 5 min and washed once with sterile PBS. Pelleted cells were suspended in 1 ml PBS containing 500 U hyaluronidase and incubated for 1 hour at 37°C. The pellet was washed 2 times with ice cooled PBS and 2 times with ice cooled sterile sucrose (0.625 M). Subsequently, the pellet was resuspended in 1.5 ml sucrose (0.625 M) and 100 μl were aliquoted in 1.5 ml Eppendorf tubes. The competent cells were stored at -80°C.

### RNA isolation

Total RNA from GAS strains was isolated from 25 ml of culture at the mid-exponential phase of growth by the usage of FastRNA Pro Kit (MP Biomedicals). In brief, the bacterial pellet was resuspended in 1 ml RNApro solution, transferred to a lysing matrix tube and processed through a Hybaid RiboLyser instrument for 40 seconds at setting of 6.0. After centrifugation, the lysate was subjected to chloroform extraction. The upper phase was mixed with absolute ethanol and incubated at -20°C for 2 hours. After washing with 70% ethanol, the RNA pellet was dried and resuspended in DEPC-H_2_O.

### First-strand cDNA synthesis and reverse transcription PCR (RT-PCR)

DNAse digestion of the obtained RNA was carried out with RNeasy-Free DNase Set (Qiagen). After DNase treatment, 5 μg of total RNA was used for first-strand cDNA synthesis with SuperScript™ II reverse transcriptase using random primers (Invitrogen) according to the manufacturer's instructions. For the RT-PCR analysis two pairs of primers were used binding to *covR *and *covS*, correspondently. A fragment with size of 625 bp appears when using primers binding to *covR*, CovR_for (5'-CTCTTGAGCTGCAACATGAGG-3') and CovR_rev (5'-CACGAATAACGTATCCCATGC-3'). A PCR employing primers binding to *covS*, CovS_for (5-ATCATCTCCTGGCTTGCATGG-3') and CovS_rev2 (5'-CCAGTCACTGAAAGGTTAATCGC-3'), results in a product with a size of 846 bp. As controls genomic DNA and total RNA were used as template for the PCR analysis with both primer pairs.

### Construction of recombinant vectors and GAS mutants

**Figure 1:**
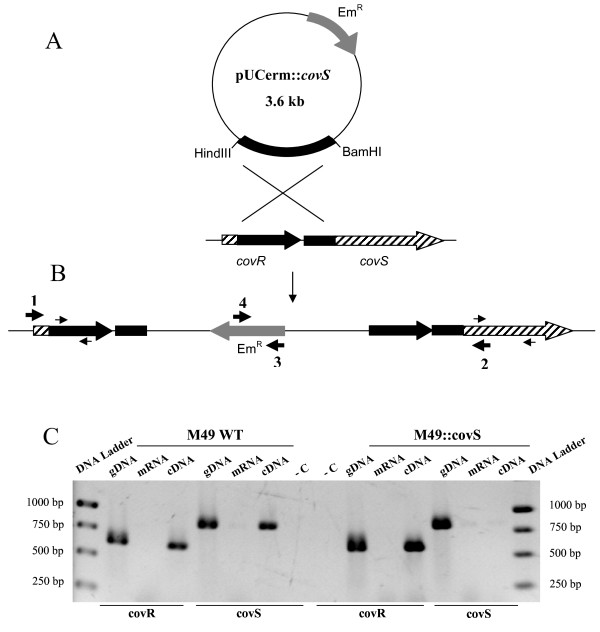
**Schematic representation of the inactivation of CovS**. A. Inactivation of CovS in the serotype M49 strain 591. Plasmid pUCerm::*covS *contains a fragment internal of *covRS *and confers erythromycin resistance (Em^R^). The genomic regions from both *covR *and *covS *used for recombination are marked in black. B. M49 *covRS *locus after insertion of the plasmid pUCerm::*covS*. The thin arrows depict primers used for RT-PCR analysis (see below). The thick numbered arrows (1-4) represent primers used for PCR of whole region and junction fragments to confirm plasmid integration into the chromosome. C. RT-PCR analysis. Primer pairs derived from *covR *and *covS *were used. Lane DNA Ladder, O'GeneRuler 1 kb DNA Ladder (Fermentas); gDNA, genomic DNA; cDNA, first-strand synthesized cDNA; mRNA, messenger RNA; -C, negative control, where no template for polymerization was used.

Using *S. pyogenes *M49 chromosomal DNA as a template, a 1023 bp internal fragment of *covRS*, spanning 663 bp from the *covR *gene and 355 bp from *covS *gene, was amplified by PCR employing primers Csrko_for_HindIII (5'-GGCGGC**AAGCTT**GAAGATGAAAAGAATCTGG-3') and Csrko_rev_BamHI (5'-GGCGGC**GGATCC**AGACATAAATATCTTGATTCG-3'). The internal fragment was digested by HindIII and BamHI and subsequently cloned into pUCerm [24] to obtain a plasmid designated pUCerm::*covS*. For electroporation, thawed electrocompetent cells (100 μl) were initially mixed on ice with 10 μl pUCerm::*covS*. The mixture was next transferred to a pre-chilled 2 mm electrode spacing cuvette (Bio-Rad). Electroporation was then performed using a Gene Pulser II electroporator (Bio-Rad) with the following settings: voltage 1750 V, capacitance 25 μF, 12 ms, 481Ω. Subsequently, 1 ml pre-warmed Todd-Hewitt broth (Invitrogen) supplemented with 0.5% yeast extract and 0.125 M sucrose was added to the transformed cells, and the suspension was incubated at 37°C for 2 h. Transformants were selected on THB agar plates supplemented with 0.5% yeast extract and 5 μg/ml erythromycin. Successful integration of the plasmid was confirmed by PCR analysis of junction fragments using standard protocols (for the used primer locations please refer to fig. 1 and the result section). The generated insertional mutant strains were designated M18::*covS*, M18_588::*covS*, M49::*covS*, M49_581::*covS*, M49_634::*covS*, M2::*covS*, M2_583::*covS*, M6::*covS*, M6_586::*covS*, M6_796::*covS *and M6_576::*covS*.

From each GAS serotype under investigation a WT and mutant strain pair was tested for unaltered growth phenotypes in regular batch cultures using THY and BHI medium (additional file 1)

### Eukaryotic cell adherence

For all adherence studies the HaCaT cell line was used, which is a spontaneous immortalized human keratinocyte cell line [25], obtained from German Cancer Research Center, Heidelberg, Germany. The adherence assay was performed as described previously [26]. In brief, all GAS strains were grown in THB supplemented with 0.5% yeast extract at 37°C under a 5% CO_2 _-20% O_2 _atmosphere. After overnight incubation the bacterial cells were suspended in modified Eagle's medium supplemented with 10% fetal calf serum and added to 3.5 × 10^5 ^per ml HaCaT cells grown overnight to confluence. After 2 h, the eukaryotic cells were washed with PBS and subsequently detached by adding 200 μl 0.25% trypsin/0.5 mM EDTA for 10 min at 37°C. To quantify bound bacteria, the cells were lysed with distilled water and the number of bacteria in the lysate was assessed by viable counts.

### Biofilm assays

Biofilm phenotype formation was studied under static conditions using uncoated or fibronectin-coated (for M49, M2, M6 serotype strains) and collagen I-coated (for M18 serotype) polystyrene well plates. BHI (brain heart infusion), supplemented with 0.5% (w/v) glucose was used for all biofilm experiments. This medium was shown to best support primary GAS adherence and biofilm formation in a previous study from our lab [17]. For quantitative measurements, safranin staining was performed as previously described [17]. For the SEM studies biofilms were grown on coverslips coated with human collagen I (Biomol) and further processed as described by Lembke et al. [17].

### Capsular hyaluronic acid measurements

The amount of cell-associated hyaluronic acid produced by each GAS strain was determined by releasing capsule from exponential-phase GAS cells grown in THY and measuring the hyaluronic acid content of the cell extracts using 1-ethyl-2-[3-(1-ethylnaphtho[1,2-d]thiazolin-2-ylidene)-2-methylpropenyl]naphtho[1,2-d]thiazolium bromide (Stains-all, Sigma) as described previously [27]. Absorbance values were compared with a standard curve generated using known concentration of hyaluronic acid from *Streptococcus equi *and the amount of hyaluronic acid capsule produced from the tested strains was expressed as femtograms (fg) per colony-forming unit (CFU).

### Blood survival assay

The blood survival assay was carried out as described by Nakata et al. [21]. Briefly, wild type and CovS mutant strains were grown to exponential growth phase. The bacteria were harvested by centrifugation and set to an optical density at 600 nm of 0.25. This suspension was further diluted 1:10000 in PBS. After determination of CFU in the suspension, 20 μl of it was incubated together with 480 μl of heparinized blood for 3 hours at 37°C with rotation. Finally, the remaining CFU were determined and related to the initial inoculum, which was set to 100%.

### Statistical analysis

A statistical analysis for all functional tests was performed by two-tailed paired Student's t test.

## Results

### Inactivation of CovS in GAS serotypes

CovS deficient mutants were constructed in different GAS serotype strains by insertional mutagenesis. By this technique, the *covS *gene is physically separated from its promoter, thereby blocking transcription and thus expression of the CovS protein. (Fig. 1). A plasmid pUCerm::*covS *containing a HindIII-BamHI fragment derived from *covS *and *covR *sequences from M49 strain 591 was used (Fig. 1A). Comparative sequence analysis of the full genome sequenced serotypes, carried out in order to evaluate the degree of homology between *covRS *sequences among different GAS serotypes, revealed that *covRS *is highly conserved in GAS. By means of the BLASTN program http://blast.ncbi.nlm.nih.gov/Blast.cgi, the identity rate between the nucleotide sequences of CovRS from various GAS serotypes was determined to be at least 99%. Therefore, the construct containing an internal part of the *covRS *nucleotide sequence derived from M49 serotype genome was used for insertional inactivation of *covS *in multiple serotypes. The resulting erythromycin resistant strains were analyzed by conventional PCR for verification of the inactivation of *covS*. As shown in Fig. 1B, the conventional PCR was performed with primer pairs 1/2, 1/3, and 4/2 and products with the expected fragment sizes were received (data not shown). As expected, primer combinations 1/3 and 4/2 did not give any fragments using WT chromosomal DNA as template (data not shown). Furthermore, to assure that transcription of *covS *does not occur in the inactivated strains, RT-PCR analyses were carried out. As shown in Fig. 1C, when using primers derived from *covR *and cDNA as a template, both the wild type M49 strain and its correspondent mutant strain gave a band of 625 bp. However, PCR employing primers from *covS*, showed a signal with a size of 846 bp only when cDNA isolated from the M49 wild type, but not from the M49::*covS *mutant strain was used.

To exclude the possibility of general growth defects in the mutants under the experimental conditions tested we performed regular batch cultures and monitored the growth by optical density readings at OD_600 _nm in hourly intervals. Exemplary results for one WT/mutant pair from each serotype are shown in additional file 1. No general growth defects were observed for growth in THY and BHI (additional file 1).

### Contribution of CovS to biofim formation

Apart from primary adherence to eukaryotic cells, it is now evident that GAS can form biofilms on matrix protein-coated and uncoated surfaces [17]. Our previous work investigating the contribution of different TCSs to biofilm phenotype formation suggested CovRS to be involved in biofilm formation in GAS (unpublished observations). Work from Cho and Caparon has also suggested that CovRS activity is required for biofilm formation [18]. Thus, we performed extensive biofilm studies with wild type strains from different serotype strains and their correspondent CovS mutant strains.

Previously, Lembke et al. showed that GAS serotypes preferentially adhered to human matrix-protein-coated surfaces. For instance, collagen type I was described as the matrix protein supporting to the highest extent the primary adhesion of M18 GAS serotype. Fibronectin coating was reported to induce biofilm formation in M2 and M6 and even in the biofilm-negative serotype M49 [17]. Based on these observations, collagen type I or fibronectin was used as a coating protein when M18 or M49, M2 and M6 biofilm phenotypes were studied, respectively.

**Figure 2:**
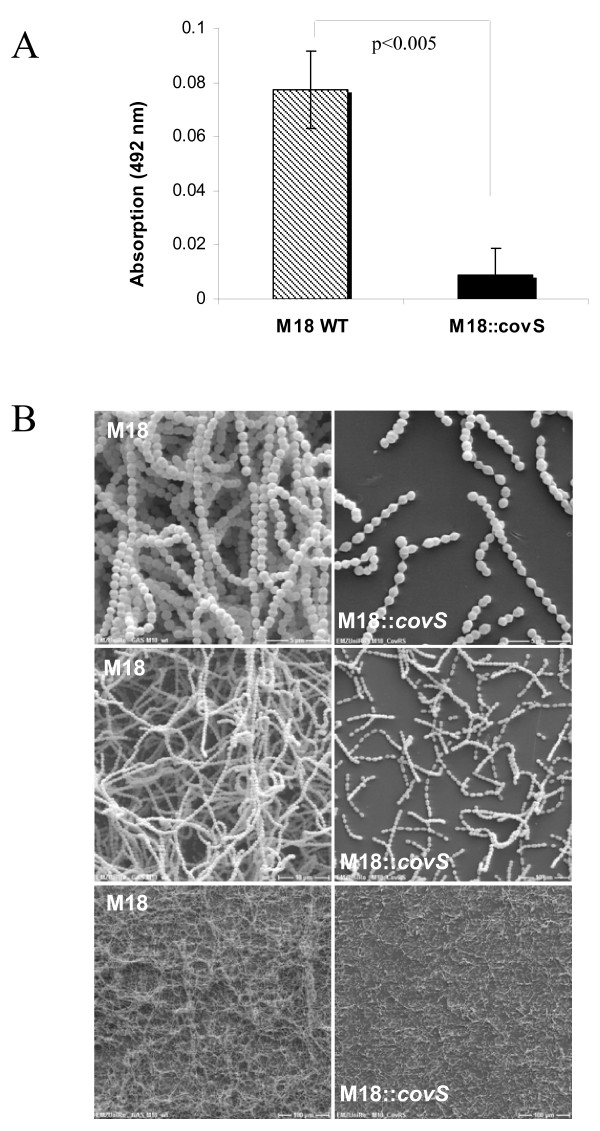
**Biofilm production of serotype M18 GAS and M18::*covS *mutant strains**. The GAS strains were grown on a polystyrene well surface or plastic coverslips, coated with human collagen type I, for 72 h in static culture. A. Safranin assay. B. Scanning electron microscopy. Different magnifications are presented as follows: 200×, 2000×, 5000× (from lower to upper panel, respectively). The P-value of differences as determined by two-tailed paired Student's t test is shown above the columns in panel A.

**Figure 3:**
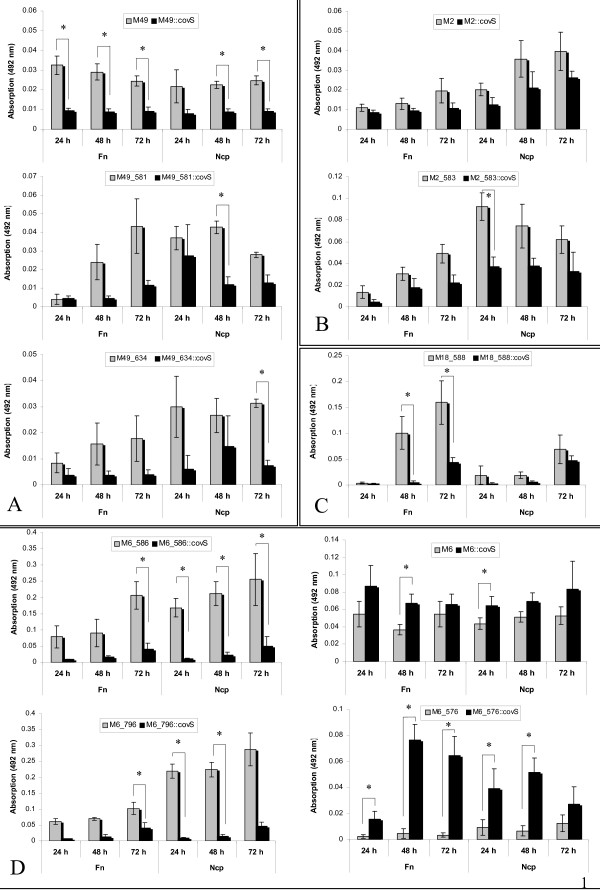
**Biofilm formation abilities of CovS mutant strains and corresponding parental strains in different GAS serotypes**. A. M49::*covS*, M49_581::*covS *and M49_634::*covS *mutants, and the correspondent wild type M49 GAS strains. B. M2::*covS *and M2_583::*covS *mutants and the correspondent wild type M2 GAS strains. C. M18_588::*covS *mutant and wild type M18_588 GAS strain. D. M6_586::*covS*, M6::*covS*, M6_796::*covS *and M6_576::*covS *mutants and the correspondent wild type M6 GAS strains. The biofilm production under static conditions in BHI media supplemented with 0.5% (w/v) glucose was quantified by safranin assay. The incubation time is presented in hours (h). The surfaces for biofilm formation were either non-coated (Ncp, no coating protein) or coated with fibronectin (Fn). Data reported represent the mean and standard error of the mean derived from three independent experiments. The significance level as determined by two-tailed paired Student's t test is indicated (*).

As shown in Fig. 2A the inactivation of CovS sensor kinase expression in the M18 serotype led to biofilm defective mutants. This observation was further confirmed by SEM analysis (Fig. 2B). A similar phenotype of biofilm defectiveness was observed for the other CovS mutant GAS serotype strains irrespective of using none-coated or fibronectin-coated polystyrene surfaces (Fig. 3). Inactivation of CovS expression in the M49 serotype background resulted in a biofilm-negative phenotype (Fig. 3A). Even when human fibronectin was used as a matrix protein surface coating, the CovS M49 mutant strain was still defective in biofilm production. Likewise, the M2::*covS*, M2_583::*covS *and M18_588::*covS *mutant strains were attenuated in their biofilm-forming capacity in contrast to the corresponding parental strains (Fig. 3B and 3C).

Since it was previously shown that the CovRS sytem is a negative regulator of hyaluronic acid capsule synthesis [5] and because of the fact that the capsule is involved in biofilm formation or maturation [18], it was unexpected that inactivation of CovS in this study prevented the biofilm production. However, our results clearly demonstrated that the CovS mutants in the M18, M49 and M2 serotype are defective in biofilm formation in comparison to the respective wild type strains.

Of note, for two out of the four M6 serotype strains used in our study, the ability of the CovS mutant to form biofilm exceeded that of the wild type M6 strain. As shown in Fig. 3D the strains M6_576::*covS *and M6::*covS *showed an increased biofilm phenotype. In contrast, the inactivated CovS strains M6_586::*covS *and M6_796::*covS *displayed a biofilm-deficient phenotype. In addition, Cho and Caparon reported that inactivation of CovRS in another *S. pyogenes *M6 resulted in a failure of biofilm formation [18]. Therefore, our results could be indicative of a strain-dependent CovS influence on the GAS biofilm formation abilities in the M6 genetic background.

### Contribution of CovS to capsule formation in GAS

To reveal if the observed heterogeneity in the biofilm formation abilities of the generated CovS mutants correlates to capsule synthesis and to further evaluate the serotype-dependent contribution of CovS to capsule formation, a quantitative analysis of capsule expression was performed. The GAS capsule is an important virulence attribute, shown to be associated with enhanced resistance to phagocytic killing in vitro and with increase in virulence [5]. The capsule is involved in attachment of GAS to the hyaluronic-binding protein CD44 on pharyngeal epithelial cells [28]. Furthermore, capsular hyaluronic acid of GAS hampers their invasion into human pharyngeal epithelial cells [29].

The capsule measurements revealed that the ability of the tested strains to form capsule differs. M18 strains produced the highest amount of hyaluronic acid capsule whereas the clinical isolate 591 M49 strain behaved as a low capsule producing strain. However, as shown in Table 1, for all of the strains, the amount of capsule detected in the correspondent CovS mutants was increased in comparison with the parental wild type strains. Even though the extent of increment of capsule synthesis of CovS inactivated mutants differs among the tested strains, our results suggest that repression of capsule synthesis is a uniform feature of the CovS sensor kinase across GAS serotype borders. Furthermore, the capsule formation cannot explain the divergent effect of CovS inactivation on biofilm phenotype in different GAS strains as the capsular hyaluronic acid measurements revealed that the M6::*covS *inactivated mutant overproduced capsule similarly to all other tested serotypes (Table 1).

**Table 1:** Capsular hyaluronic acid measurements.

Strains	Capsule-associated hyaluronic acid (fg/CFU)
M49	14.0 ± 1.5
M49::covS	38.6 ± 3.6
M18	87.2 ± 0.2
M18::covS	114.7 ± 3.7
M2	15.5 ± 3.6
M2::covS	30.6 ± 3.3
M6	15.5 ± 1.7
M6::covS	23.9 ± 0.2

Hyaluronic acid amounts produced by the GAS strains used was determined as described previously [27] and was expressed as fg of hyaluronic acid per CFU.

### Contribution of CovS to adherence of GAS

**Figure 4:**
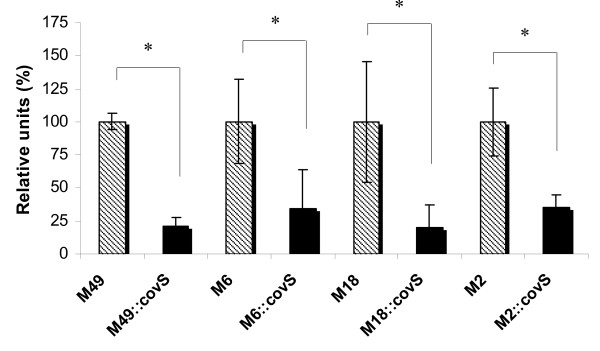
**Adherence to HaCaT cells**. The adherence of CovS mutant strains is presented as a percentage of the data determined for the corresponding parental strains. The data represent the mean values of three independently performed experiments. *, the significance level (p < 0.05) for differences between wild type and isogenic mutant strains was determined by two-tailed paired Student's t test.

We next tested the serotype-dependent contribution of CovS to adherence to human keratinocytes (HaCaT cell line [25]). Adherence abilities of the CovS mutants in comparison with the corresponding wild type serotype strains are shown in Fig. 4. The results are presented as relative percentages, where the wild type adherence was set to 100%. For all examined serotypes the inactivation of CovS led to a reduction in the adhesion rate to human keratinocytes. The observation that highly encapsulated mutant CovS strains are attenuated in keratinocyte attachment suggested that the capsule might prevent the interaction of bacterial surface molecules with specific receptors on keratinocytes by blocking the function of different adhesins through physical shielding. A similar finding was made previously by Darmstadt and co-workers, who reported that hyaluronic acid capsule impedes the interaction of bacterial adhesins with the keratinocyte receptor [30]. The adherence of a mutant lacking hyaluronic acid capsule (*has *mutants) was increased 13-fold [30]. Furthermore, Schrager and others pointed out that acapsular GAS exhibit enhanced adherence to human keratinocytes [28]. Therefore, we assume that CovS inactivation in different serotype GAS strains led to reduction in the adherence ability of the mutant strains in comparison with the corresponding wild type strains, which might be explained by the overexpressed capsule in the CovS defective mutants. However, the CovS influence on keratinocyte adherence among the tested GAS serotypes is apparently a uniform feature.

### Contribution of CovS to survival of GAS in whole blood

**Figure 5:**
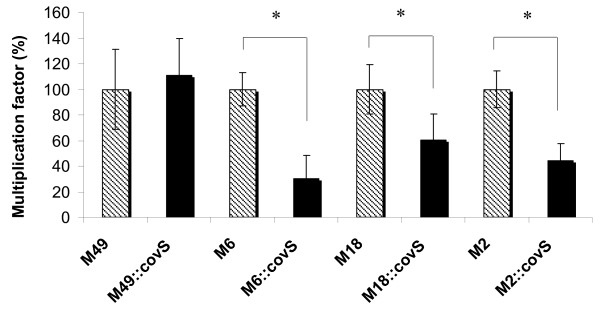
**Survival of wildtype and CovS mutants in whole blood**. The multiplication factor for each CovS mutant strain is shown as percentage from the data obtained with the corresponding wild type strain, which was set to 100% for each independent test. The data represent the mean values and standard deviations from two independent sets of experiments using blood from three different donors. *, indicates significance level for differences between wildtype and isogenic mutant strains as calculated by the two-tailed paired Student's t test.

GAS are known to be very well equipped for survival in whole human blood by expression of a diverse armamentarium of virulence factors that interfere with primary host defense mechanisms in the blood, in particular the complement system and phagocytosis [17]. Increased capsule expression leads to mucoid strains that are very often more virulent compared to unencapsulated strains [31] and have an increased resistance to phagocytic killing [1]. Thus, exponential-phase wild type and CovS mutant strains were tested for survival in whole human blood. As shown in Fig. 5, mutation of CovS in GAS serotypes M2, M6 and M18 leads to a significantly reduced ability of the strains to survive and multiply in blood. This finding was unexpected since the increase in capsule amounts should allow for a better survival and multiplication. However, many other GAS surface-associated and secreted virulence factors have been described to act as defense against phagocytic killing [4,32] and some of them might be more dominant in their protective effect compared to capsule. At least for M18 it was shown that capsule may be responsible for phagocytosis resistance in serum, whereas survival in blood to a larger extend relied on M protein expression [33]. Lack of CovS protein expression had no effect on blood survival of the GAS M49 serotype (Fig. 5) suggesting that the influence of CovS on blood survival is not a uniform feature among different GAS serotypes.

## Discussion

Lately, an increasing amount of data compile to a strong argument for a strain-dependent transcriptional regulation in GAS. In addition, a comparison of the set of genes regulated by CovRS in different *Streptococcus agalactiae *(Group B streptococci, GBS) strains revealed variations in their CovRS regulons. Thus, a strain-specific manner of CovRS-mediated gene regulation in GBS was reported [34].

In this study, we investigated the potential effect of the sensor kinase CovS on virulence traits of different *S. pyogenes *serotypes strains, in order to figure out if a serotype- or strain-dependent influence of CovS regulation in GAS does occur in the genetic background of an intact response regulator CovR.

Although CovRS has been described as a global regulatory system in GAS, our results clearly showed that variations of the CovS effect on biofilm formation appear and that strain-dependent diversity in the CovRS regulons might exist also in GAS. Biofilm production has been described recently as an important protective mechanism of GAS associated with increased antibiotic resistance [17]. Previously, Cho and Caparon [18] showed that a M6 mutant lacking CovR failed to form biofilms, which suggests that the CovRS TCS is required for biofilm formation in GAS. Surprisingly, in contrast to the latter observation, our investigation showed that the CovS inactivation in other M6 strains did not lead to the same result. This statement implied that CovS might be involved in a strain-dependent influence on biofilm formation. Alternatively, since our mutant is deficient of CovS, but not CovR, the observed contradiction could be indicative of divergent influence of the response regulator CovR and histidine kinase CovS on biofilm formation. Another explanation supported by a previous study by Dalton and Scott suggested a direct or indirect influence of CovS on CovR under mild stress conditions [35]. Such stress conditions could have an influence on *S. pyogenes *biofilm growth. Further experiments presented here on the biofilm production of different GAS serotypes strains showed that the CovS influence on biofilm of GAS is a strain-dependent characteristic. This heterogeneity among different isolates could be associated with adaptation to diverse host environments. Similar findings of strain-specific differences between transcriptional regulatory networks in GAS were made recently with the Nra regulator, which controls the transcription of several key virulence factors in GAS [20,36,37]. In contrast to the M49 strain, where Nra acts as a negative regulator of pilus gene transcription, Nra functions as a positive regulator of pilus gene transcription in an M53 strain [20].

As already mentioned the hyaluronic acid capsule is an important virulence factor, required for resistance to complement-mediated phagocytic killing and thus is associated with enhanced virulence [1,27,38,39]. Previous investigations showed that acapsular mutant strains of GAS were impaired in pharyngeal colonization ability [38]. In contrary, highly encapsulated or mucoid strains have been linked to acute rheumatic fever and severe invasive infections [5]. Various studies on regulation of capsule expression revealed that the regulatory protein Mga, shown to influence the expression of diverse GAS pathogenicity factors, affects the hyaluronic acid synthesis in GAS in a serotype- or strain- dependent mode. For instance, inactivation of Mga showed no effect on capsule production in an M6, M18 and M49 strain, but it resulted in decreased *has *operon transcription in a M1 strain [5]. However, as our results showed, the CovS- influenced depression of capsule formation in GAS is a uniform feature among divergent GAS serotypes tested. Moreover, our results confirm previous experiments from Bernish and van de Rijn (1999) who showed that a non-polar inactivation of CovS in 3 unencapsulated strains rendered those strains highly mucoid [40].

The ability of *S. pyogenes *to adhere to its eukaryotic target cells is an essential factor both for causing disease and for persisting in its human host [16]. Therefore, the contribution of CovS to the adherence capacity of GAS in a serotype-dependent manner was additionally investigated. The results clearly showed that irrespective of their individual adherence abilities, the CovS inactivated mutants were inhibited in their adherence to human keratinocytes in comparison with the corresponding parental wild type strains. Together with the fact that the hyaluronic acid masses of CovS mutant strains exceeded those detected for the wild types, this could imply that the increased capsule material in the mutants could mask the exposure of important proteins involved in cell attachment and thus inhibit the process of attachment. Alternatively, CovS could act on important bacterial host cell adhesins either direct or via its influence on CovR.

Furthermore, the effect of depression in adherence rate typical for the CovS- inactivated mutants was observed in all the serotype tested, which suggests that CovS influences the adherence of GAS in an unvarying mode.

Of note, our data for the adherence capacity of CovS- inactivated GAS mutants contrasts the observation made for GBS, where a corresponding CovRS mutant exhibited increased adherence to epithelial cells [41,42]. Apparently, although the proteins of the CovRS signaling system are quite conserved among these two phylogenetically related species, the influence of CovS to the adhesive abilities of these two bacterial pathogens is strikingly different. However, this is not surprising, as similar heterogeneity in the transcription regulation might exist even among different strains within the same species.

Finally, CovRS has been reported to obviously respond to so far unknown molecular signals in human blood. Analysis of GAS global transcription during ex vivo culture in human whole blood revealed that CovRS is involved in GAS adaptation allowing growth in blood [13]. We observed that *covS *insertional mutants in the M6, M2 and M18 background were significantly attenuated in their efficiency to multiply in whole human blood, suggesting a high importance of the sensor kinase activity for blood survival. However, this cannot be postulated for M49 591, which is a skin isolate. Moreover, since the adaptation in human blood is associated mainly with pathogenesis during invasive growth, the involvement of CovS to the response to human blood exposure is not a uniform characteristic among different GAS serotype strains.

Most recently, a paper published during the review process of this work by Trevino and colleagues uncovered that CovR retains some regulatory activity in the absence of a functional CovS sensor kinase and that CovS mutants are hypervirulent in ex vivo and in vivo models of invasive infection [14]. However, CovS mutants were attenuated in their ability to survive in human saliva, which could be one possible explanation why no natural CovS mutants are transmitted from host to host [14].

## Conclusion

Taken together, no serotype-dependent contribution on regulation of capsule expression and adherence to human keratinocytes was observed. Interestingly, an increased capsule expression in M2, M6 and M18 CovS mutants did not lead to enhanced survival of the bacteria in whole human blood. In contrast, the effect of CovS on biofilm formation depended on the examined strain. This finding implies that the CovRS system has divergent effects on similar target genes in different strains. Thus, the CovRS system could differ with respect to its repertoire of regulated genes in a strain-dependent manner. In summary, in addition to Nra, the CovRS system is the second regulator in GAS with serotype- or even strain-dependent activity, further supporting the emerging scheme of divergent regulatory circuits in GAS.

## Authors' contributions

VS and BK designed this study. VS, RA, and CR performed the research. VS, TF, AP, and BK analyzed the data and wrote the paper. All authors read and approved the final manuscript.

## Supplementary Material

Additional file 1 **Growth kinetic curves of the CovS mutant and corresponding parental strains**. This file depicts the growth of one WT/mutant pair from each serotype in both BHI medium (A) as well as THY medium (B). Growth was monitored by optical density measurements at OD_600 _nm in hourly intervals. Click here for file
